# Prevalence of Subclinical Hypothyroidism among Patients with Acute Myocardial Infarction

**DOI:** 10.5402/2011/810251

**Published:** 2011-07-28

**Authors:** Okuyan Ertugrul, Uslu Ahmet, Enhos Asim, Hepgul E. Gulcin, Ayca Burak, Avsar Murat, Yildiz S. Sezai, Halil Ibrahim Biter, Dinckal M. Hakan

**Affiliations:** ^1^Cardiology Clinic, Bağcılar Education and Research Hospital, Bagcilar, 34200 Istanbul, Turkey; ^2^Internal Medicine Clinic, John F Kennedy Hospital, 34100 Istanbul, Turkey; ^3^General Surgery Clinic, Bağcılar Education and Research Hospital, 34200 Istanbul, Turkey

## Abstract

*Introduction*. Subclinical hypothyroidism (SCH) is defined as a serum thyroid-stimulating hormone (TSH) level above the upper limit of normal despite normal levels of serum free thyroxine. There is growing evidence that SCH is associated with increased cardiovascular risk. We tried to investigate prevalence of SCH in acute myocardial infarction patients. *Methods and Results*. We evaluate free T3, free T4, and TSH levels of 604 patients (age 58.4) retrospectively, who have been admitted to the coronary intensive care unit between years 2004–2009 with the diagnosis of ST elevation (STEMI) or non-ST elevation acute myocardial infarction (NSTEMI). Mild subclinical hypothyroidism (TSH 4.5 to 9.9 mU/l) was present in 54 (8.94%) participants and severe subclinical hypothyroidism (TSH 10.0 to 19.9 mU/l) in 11 (1.82%). So 65 patients (10.76%) had TSH levels between 4.5 and 20. *Conclusions*. In conclusion, 65 patients (10.76%) had TSH levels between 4.5 and 20 in our study, and it is a considerable amount. Large-scale studies are needed to clarify the effects of SCH on myocardial infarction both on etiologic and prognostic grounds.

## 1. Introduction

Subclinical hypothyroidism (SCH) is defined as a serum thyroid-stimulating hormone (TSH) level above the upper limit of normal despite normal levels of serum free thyroxine [1].

Subclinical hypothyroidism or mild thyroid failure is a common problem, with a prevalence of 3% to 8% in the population without known thyroid disease [2, 3]. The prevalence increases with age and is higher in women [2]. After the sixth decade of life, the prevalence in men approaches that of women, with a combined prevalence of 10%. Antithyroid antibodies can be detected in 80% of patients with SCH, and 80% of patients with SCH have a serum TSH of less than 10 mIU/L.

There is growing evidence that SCH is associated with lipid abnormalities, increasing cardiovascular risk, particularly in older women [4, 5]. Clinical hypothyroidism is associated with premature atherosclerosis and increased prevalence of coronary disease. This is at least partly due to the lipid abnormalities often found in hypothyroidism [6, 7]. Possible mechanisms behind the link between hypothyroidism and atherosclerosis, other than dyslipidemia, include the effects of thyroid hormones on coagulation, vasodilation, parasympathetic function, and homocysteine metabolism [4, 8].

A study, which examined the relation between cardiovascular disease and TSH levels in euthyroid patients, found significantly higher TSH in patients with coronary events compared to controls matched for age, gender, and body mass index [9]. The cross-sectional Rotterdam study showed an association of SCH with myocardial infarction and aortic calcification [10].

Several observational studies comparing the outcome of SCH individuals with euthyroid subjects have shown divergent results, and it has been debated for some time whether SCH is independently associated with ischemic heart disease (IHD) [11, 12]. If the latter were true, this would be an important public health issue for the aging population, in which SCH is most prevalent [2]. 

As findings of several studies support the influence of SCH on ischeamic heart disease, we tried to investigate prevalence of SCH in acute myocardial infarction patients.

## 2. Methods

We evaluate free T3, free T4, and TSH levels of 604 patients (age 58.4) retrospectively, who have been admitted to the coronary intensive care unit between years 2004–2009 with the diagnosis of ST elevation (STEMI) or non-ST elevation acute myocardial infarction (NSTEMI). Patients were classified into 2 groups based on their thyroid function tests [13], subclinical hypothyroidism: TSH > 4.50 and <20.0 mU/L with a normal FT4 (*n* = 65) and patients with normal TSH and FT4 levels. On the basis of the definitions used by the US Preventive Services Task Force [14] and on expert consensus [15], subclinical hypothyroidism was subclassified according to the following TSH levels, 4.5 to 9.9 mU/L (*n* = 54) and ≥10.0 mU/L (marked elevation, *n* = 11), because of possible greater risks above this cutoff. We collected self-reported alcohol and smoking status. All physical examination findings including blood pressure, heart rate, and body mass index were noted. Hypertension was defined as self-report and the use of antihypertensive medications or as a blood pressure ≥140/90 mm Hg. Diabetes was defined as a fasting glucose ≥120 mg/dL or the use of hypoglycemic medication. Atrial fibrillation at baseline was self-reported or determined on electrocardiogram or with a Holter monitor. For prevalent HF at baseline, self-reports were confirmed by physical examination and echocardiography.

## 3. Results

The mean age was 58.4 years. More than half of the patients (55.6%) were males (Table 1). Mild subclinical hypothyroidism (TSH 4.5 to 9.9 mU/L) was present in 54 (8.94%) participants and severe subclinical hypothyroidism (TSH 10.0 to 19.9 mU/L) in 11 (1.82%). So 65 patients (10.76%) had TSH levels between 4.5 and 20. Subclinical hypothyroidism was more common among men in our study population. However, severe SCH was more prominent among women. Age, history of DM, history of hypertension, previous MI, presence of AF, increased diastolic blood pressure, increased triglyceride levels, inhospital deaths, and history of heart failure all were statistically more prominent among SCH patients with TSH levels between 10 and 19.9 (Table 1). Inhospital deaths, history of heart failure, HT, and DM were significantly higher in overall SCH patients than patients with normal TSH levels.

## 4. Discussion

This study is a descriptive study aiming to evaluate SCH prevalence among patients with acute MI and, demographic differences between patients with and without SCH. This study does not provide any insight into the potential mechanisms of any adverse effects of SCH on coronary artery disease.

In our study, inhospital mortality was significantly higher among patients with SCH. The cross-sectional Rotterdam study showed an association of SCH with myocardial infarction and aortic calcification [10]. In contrast, the Wickham study [16] showed no increased cardiac mortality in a 20-year followup. A more recent observational study also did not show any association between unrecognized SCH and cardiovascular events or mortality [13]. However, several more recent meta-analyses of observational studies found an association between SCH and coronary artery disease [17–19]. The risk is lower when higher quality studies are pooled [18]. A recent analysis of 7 cohort studies concluded that the relative risk of all-cause mortality was increased compared with euthyroid controls, particularly in patients with comorbid conditions [20]. Another meta-analysis of 15 studies showed an increased prevalence and incidence of cardiovascular mortality only in a relatively younger population [21]. Taken together, the findings of these 6 recent meta-analyses suggest that a cardiovascular risk exists for persons younger than age of 70 years with no effect for those aged 70 to 80 years and a possibly protective effect for those older than 80 years [22]. Thus, the cardiovascular risk issue remains controversial, and large-scale, multicenter, randomized, placebo-controlled studies are needed to assess the efficacy of levothyroxine therapy in risk reduction. 

Because of its high prevalence in the population and its adverse effects on lipid profile and hence increased cardiovascular risk, hypothyroidism is the principal functional disorder. Lipid anomalies associated with hypothyroidism are at least partially responsible for the increase in coronary heart disease. In our study, the lipid profiles of patients with and without SCH were similar. Only triglyceride levels were significantly higher in patients with TSH levels between 10–19.9 than euthyroid patients. Lipid abnormalities vary greatly between individuals, and the relationship between SCH, dyslipidemia, and cardiovascular risk is still the subject of debate [1]. The Colorado Health Fair study showed that the mean total cholesterol level was 216 mg/dL for euthyroid patients and 224 mg/dL for patients with SCH [23]. Several randomized studies have shown reduction of low-density lipoprotein cholesterol by levothyroxine therapy. However, most of the studies showing benefit are not categorized for serum TSH levels of 5.0 to 10.0 mIU/L. A meta-analysis of 13 studies concluded that the lipid profile improved with therapy [24]. In a 2004 review, data were considered insufficient to show benefits of levothyroxine therapy on lipid levels [15].

Thyroid hormones modulate enzyme activity, receptor expression, and lipid breakdown and clearance, thereby contributing to the expression of the lipid phenotype. The spectrum of thyroid dysfunction is associated with different levels of cardiovascular risk, as demonstrated by independent changes in the severity of risk factors that have been linked to cardiovascular morbidity in many studies. The increase in cardiovascular risk, most clearly associated with hyper- and hypothyroidism, is due not only to alterations in lipid profile, but also to hemodynamic changes, endothelial dysfunction, coagulation disturbances, hormonal and metabolic changes, and changes in measurable factors such as homocysteine and C-reactive protein, which are known to increase risk for atherosclerotic disease.

Several authors support the early treatment of SCH. We conclude that further studies are required to clarify the association between clinical and subclinical alterations in thyroid function, particularly the effect of various degrees of SCH on lipoprotein oxidation, in the onset and progression of atherosclerosis, and hence cardiovascular morbidity.

There have been suggestions that age and gender may have an impact on IHD risk in people with SCH. In our study population, patients with SCH were older than euthyroid group. This might also have impact on mortality and risk profile of this patient group.

Currently, the indications for therapy of SCH in the general population are unclear. However, there is some evidence that adverse vascular risk factors may be ameliorated by L-T4 treatment [25–29]. In contrast, the effect of L-T4 therapy on symptoms and well-being in SCH remains ill defined [25–28]. If therapy of SCH is to be considered on prognostic grounds, the finding that SCH is a more marked vascular risk factor in younger patients may have substantial implications. Several individual studies have demonstrated that SCH in advanced age may not be associated with an adverse prognosis [13, 30]. Thus, there may be an age or risk threshold, which remains to be defined, above which SCH should no longer be considered for treatment on prognostic grounds alone. Only well-powered prospective randomized studies with age-stratified groups, and vascular events as the primary endpoint rather than surrogate markers, will give clear answers to this complex question. Before recommending routine screening of the general population, large-scale randomized trials are needed to prove that treatment will improve quality of life in otherwise healthy patients who have the mildly elevated TSH level (5–10 mIU/L) typical of most SCH cases. But authors of this paper believe that clinicians should have a low threshold for obtaining a serum TSH level in patients with high risk for MI and atherosclerotic heart disease.

There is biological plausibility to the premise that subclinical thyroid dysfunction may cause adverse cardiac consequences. Thyroid hormone has both inotropic and chronotropic effects, and it is clear from studies of individuals with overt thyroid disease that hyperthyroidism leads to increased heart rate, increased cardiac output, and decreased systemic vascular resistance, whereas hypothyroidism has the opposite effects. In the continuum of thyroid dysfunction, therefore, it is reasonable to propose that a dose-response effect exists, with more subtle cardiac impairment being present in less extreme degrees of thyroid dysfunction. The key challenges are measuring these more subtle cardiac effects and, more importantly, determining their clinical relevance. Addressing these issues requires larger numbers of study participants and more precise measurement of the cardiac phenotype than in overt thyroid disease. It also requires careful consideration of the age and underlying cardiac status of the population studied. Mild perturbations in circulating thyroid hormone levels could either provide a tipping point for older individuals with marginal cardiac reserve or have negligible effects in the face of stronger, competing risk factors for cardiac dysfunction.

Thyroid hormone has many effects on cardiovascular hemodynamics, such as heart rate, cardiac output, systemic vascular resistance, and blood pressure. Hypothyroidism is associated with decreased endothelium-dependent vasodilatation, and animal studies have proposed that the thyroid status alters the capacity for both formation and response to nitric oxide. Endothelial dysfunction in patients with SCH may result from reduction in nitric oxide availability, with resultant impairment of flow-mediated vasodilatation [15, 31].

Recent studies suggest that there is evidence of inflammation and endothelial dysfunction in hypothyroidism. Soluble CD40 ligand (sCD40L) is a protein expressed mainly by activated platelets which have been found to be associated with cardiovascular events. In a recent study, it has been shown that patients with overt and subclinical hypothyroidism associated with chronic autoimmune thyroiditis had decreased serum levels of sCD40L [32].

Studies have shown slowed left ventricular relaxation time, increased vascular tone at rest, and left ventricular systolic dysfunction with exercise and impaired endothelial function [33]. Some studies have shown improvement of cardiac contractibility and systolic time interval with levothyroxine therapy [33]. No evidence exists to support an association between heart failure and a serum TSH level of less than 10.0 mIU/L. Again, most studies were not categorized for degrees of TSH elevation, and data remain insufficient for a TSH level less than 10 mIU/L but strongly suggestive for a TSH level greater than 10 mIU/L.

A double-blind, placebo-controlled trial examining the relation between thyroid function and another risk factor for atherosclerotic disease—CRP—found significantly higher concentrations in patients with clinical and subclinical hypothyroidism, and CRP levels rose further with worsening thyroid failure [34].

There is conflicting evidence concerning the effect of hypothyroidism on coagulation. Both increased and decreased platelet aggregation have been reported, with the degree of severity determining the impact on coagulation parameters [35, 36]. In moderate hypothyroidism (TSH 10–50 mU/L), fibrinolytic activity is reduced, with low d-dimers, increased *α*2-antiplasmin activity, and higher levels of antigens to tissue plasminogen activator (t-PA) and plasminogen activator inhibitor-1 (PAI-1) [36]. In severe hypothyroidism (TSH > 50 mU/L), d-dimer levels are increased and a 2-antiplasmin activity and t-PA and PAI-1 antigen levels are reduced [36]. These findings suggest that there is greater risk of thrombosis, and hence of myocardial infarction, in moderate hypothyroidism, and greater risk of hemorrhage in severe hypothyroidism.

In conclusion, 65 patients (10.76%) had TSH levels between 4.5 and 20 in our study, and it is a considerable amount. Large-scale studies are needed to clarify the effects of SCH on myocardial infarction both on etiologic and prognostic grounds.

## Figures and Tables

**Table 1:** Baseline characteristics of the patients according to thyroid status. (Values are mean ± SD percentages. *P < 0.05 compared with euthyroid category.)

	Total (*n* = 604)	All subclinical hypothyroids (*n* = 65)	Subclinical hypothroid (TSH = 4.5–9.9), (*n* = 54)	Subclinical hypothyroid (TSH = 10–19.9), (*n* = 11)
Age (years)	58.4	59.9	59.4	64.2*
Sex (male/female)	336/268	41/24	37/17	4/7
Active smoker	107 (17.7%)	8 (12.3%)	7 (12.9%)	1 (9%)
Alcohol	109 (18%)	7 (10.76%)	6 (11.1%)	1 (9%)
Diabetes mellitus	103 (17%)	17 (26.15%)*	13 (24%)	4 (36.3%)*
Hypertension	296 (49%)	37 (56.9%)*	29 (53.7%)	8 (72.7%)*
Previous MI	62 (10.2%)	11 (16.9%)	7 (12.9%)	4 (36.3%)*
Atrial fibrillation	71 (11.7)	10 (15.38%)	7 (12.9%)	3 (27.2%)*
BMI (KG/M^2^)	28.2	29.4	29.3	29.7
Systolic BP	138.4	141.5	141.3	142.5
Diastolic BP	73.2	76.4	75.7	81.4*
Total cholesterol (mg/dL)	227	232	229	243
LDL cholesterol (mg/dL)	139	141	140.5	143
HDL cholesterol (mg/dL)	45	44.5	44	46
Triglycerides (mg/dL)	173	176	175	189*
Fasting blood glucose (mg/dL)	109	112	111	117
Creatinine (mg/dL)	1.01	1.0	0.98	1.1
ST elevation MI	273 (45.1%)	35 (53.8%)	29 (53%)	6 (54.5%)
Non-ST elevation MI	331 (54.9%)	30 (56.2%)	25 (47%)	5 (45.5%)
TSH levels (Mu/L)	2.97	6.8*	6.3	12.5*
Inhospital deaths	15 (2.48%)	5 (7.7%)*	2 (3.7%)	3 (27.27%)*
History of heart failure	24 (3.97%)	5 (7.7%)*	2 (3.7)	3 (27.27%)*
